# Lack of Association between Anti-Phospholipid Antibodies (APLA) and Attention Deficit/Hyperactivity Disorder (ADHD) in Children 

**DOI:** 10.1080/10446670310001626553

**Published:** 2003

**Authors:** Shay Bujanover, Yair Levy, Miriam Katz, Yael Leitner, Isaac Vinograd, Yehuda Shoenfeld

**Affiliations:** 1 Department of Internal Medicine 'B' and Center for Autoimmune Diseases Chaim Sheba Medical Center Tel-Hashomer Israel; 2 Sackler Faculty of Medicine Tel-Aviv University Israel; 3 Child Development Center Chaim Sheba Medical Center Tel-Hashomer Israel; 4 Department of Pediatrics Dana Children's Hospital Tel-Aviv Sourasky Medical Center Tel-Aviv Israel; 5 Department of Pediatric Surgery Dana Children's Hospital Tel-Aviv Sourasky Medical Center Tel-Aviv Israel

## Abstract

Numerous studies have shown the pathological influence anti-phospholipid antibodies (APLA) have on the physiology of the single neuron as well as the function of the entire human nervous system. The influence is well demonstrated in the antiphospholipid syndrome (APS). This syndrome is characterized by a triad of arterial or venous thrombotic events, recurrent fetal loss and thrombocytopenic purpura. The syndrome exhibits different neurological pathologies such as: chorea, seizures, transverse myelopathy, migraine, cerebral ataxia, hemiballismus and transient global amnesia, which are not fully explained by the procoagulopathic trait of APLA. A study on mice induced with APS demonstrated hyperactive behavior when compared to the control group. The information gathered from these different studies raised the question whether APLA has any part in the etiology of Attention Deficit/Hyperactive Disorder (ADHD) in children.

We compared 41 children diagnosed with ADHD to a control of 28 healthy children. Blood drawn from the two groups was screened using ELISA for the presence of anti-cardiolipin antibodies, antiβ2GP antibodies, anti-phosphatidyleserine antibodies and anti-ethanolamine antibodies. The results show no significant difference in the level of antiphospholipid antibodies (APLA) measured between the children diagnosed with ADHD and the control group.


*Clinical & Developmental Immunology*, June–December 2003 Vol. 10 (2–4), pp. 105–109

Taylor & Francis healthsciences

### Lack of Association between Anti-Phospholipid Antibodies (APLA) and Attention Deficit/Hyperactivity Disorder (ADHD) in Children

SHAY BUJANOVER[a,b], YAIR LEVY[a,b], MIRIAM KATZ[b,c], YAEL LEITNER[b,d], ISAAC VINOGRAD[b,e] and YEHUDA SHOENFELD[a,b,*,†]

[a]*Department of Internal Medicine 'B' and Center for Autoimmune Diseases, Chaim Sheba Medical Center, Tel-Hashomer, Israel*
[b]*Sackler Faculty of Medicine, Tel-Aviv University, Israel;* [c]*Child Development Center, Chaim Sheba Medical Center, Tel-Hashomer, Israel*
[d]*Department of Pediatrics, Dana Children's Hospital, Tel-Aviv Sourasky Medical Center, Tel-Aviv, Israel;* [e]*Department of Pediatric Surgery, Dana Children's Hospital, Tel-Aviv Sourasky Medical Center, Tel-Aviv, Israel*

Numerous studies have shown the pathological influence anti-phospholipid antibodies (APLA) have on the physiology of the single neuron as well as the function of the entire human nervous system. The influence is well demonstrated in the antiphospholipid syndrome (APS). This syndrome is characterized by a triad of arterial or venous thrombotic events, recurrent fetal loss and thrombocytopenic purpura. The syndrome exhibits different neurological pathologies such as: chorea, seizures, transverse myelopathy, migraine, cerebral ataxia, hemiballismus and transient global amnesia, which are not fully explained by the procoagulopathic trait of APLA. A study on mice induced with APS demonstrated hyperactive behavior when compared to the control group. The information gathered from these different studies raised the question whether APLA has any part in the etiology of Attention Deficit/Hyperactive Disorder (ADHD) in children.

We compared 41 children diagnosed with ADHD to a control of 28 healthy children. Blood drawn from the two groups was screened using ELISA for the presence of anti-cardiolipin antibodies, anti-β2GP antibodies, anti-phosphatidyleserine antibodies and anti-ethanolamine antibodies. The results show no significant difference in the level of antiphospholipid antibodies (APLA) measured between the children diagnosed with ADHD and the control group.

*Keywords*: Attention Deficit/Hyperactivity Disorder (ADHD); Antiphospholipid Antibodies (APLA); Antiphospholipid Syndrome (APS); Children

### INTRODUCTION

Attention Deficit/Hyperactivity Disorder (ADHD) is one of the most prevalent disorders in childhood and adolescence, with different epidemiological studies showing a prevalence of 3–5% in all school aged children in the United States (Robinson *et al.*, 1999). Almost half of all visits to neurologists, neuropsychologists and psychiatrists in this age group are ADHD related (Cantwell, 1996). Patients with ADHD display increased motor activity, inattention and impulsivity. On the long term, a large percent of these children develop psychological and developmental sequels such as: learning disability, stress disorder, mood disorder, communication and language difficulties, dyspraxia and Tourette's disorder. Although ADHD remains a heterogeneous condition for which no one etiology has emerged, there has been growing evidence for the explanation that the condition is caused by a complex interaction of biological and environmental factors. The treatments are numerous, none being definitive. The most effective is a combination of behavioral and pharmacological treatments (Elia *et al.*, 1999).

Antiphospholipid antibodies (APLA) are a diverse group of antibodies directed against proteins that are bound to phospholipids inside the cell's wall. Out of the proteins, the most investigated is β2GP and out of the phospholipids, the most investigated are: cardiolipin, phosphatidylserine, ethanolamine and phosphatidylcholine. The influence, these antibodies have on human health is well documented in the antiphospholipid syndrome (APS). This syndrome as first described by Hughes (1983), is characterized by the appearance of venous or arterial thrombosis, repeated abortions, thrombocytopenia and the existence of APLA (particularly, lupus anticoagulant and anti-cardiolipin). A wide spectrum of other clinical manifestations have been reported recently in association with APS, including

*Incumbent of the Laura-Schwartz-Kipp Chair for Autoimmunity, Tel-Aviv, Israel.
†Corresponding author. Address: Department of Medicine 'B', Chaim Sheba Medical Center, Tel-Hashomer 52621, Israel.

ISSN 1740-2522 print/ISSN 1740-2530 online © 2003 Taylor & Francis Ltd
DOI: 10.1080/10446670310001626553

valvular heart disease (Hojnik *et al.*, 1996), dermal complications and diverse neurological disorders involving mainly the central nervous system (CNS) (Brey *et al.*, 1996). The neurological manifestations presented by most patients with APS are antibody-induced coagulopathies. However, neurological manifestations other than stroke are seen in these patients, such as: chorea, seizures, transverse myelopathy, migraine, cerebral ataxia, hemiballismus and transient global amnesia, which are not fully explained by the procoagulopathic trait of APLA (Brey *et al.*, 1996).

Recent *in-vitro* studies have shown the pathological influence APLA have on neural tissue. Different research groups have demonstrated that APLA can bind to astrocytes and neurons as well as ependima and myelin cells (Kent *et al.*, 1997, 2000; Caronti *et al.*, 1998; Chapman *et al.*, 1999). The influence these have on normal neuron physiology was demonstrated by Chapman *et al.* (1999). APLA taken from patients with increased levels of APLA depolarized brain cells *in-vitro*. The *in-vivo* effect of APLA was demonstrated by Ziporen *et al.* (1997). This group induced APS on mice using monoclonal anticardiolipin antibodies. In addition to fetus resorptions and thrombocytopenia, these mice were impaired neurologically and exhibited hyperactive behavior when compared to normal control. The sum of all this gathered information raised the question whether there is an etiological connection between APLA and hyperactivity as seen in children with ADHD.

### PATIENTS AND METHODS

### Patients

In this work we compared serum samples of 41 ADHD diagnosed children to that of 28 healthy children. The work received the approval of the ethics committees of Sheba medical center, Tel-Hashmer, Israel and Sourasky medical center, Tel-Aviv, Israel. Informed consent was obtained from all parents of the subjects studied.

#### *Children with ADHD*

The patients were enrolled into the study from the patient population of the child development unit in Sheba medical center, and the pediatric neurology unit in Sourasky medical center. Each patient's file was screened to verify the diagnosis of ADHD according to the DSM IV guidelines (American Psychiatric Association, 1994). The picked age range was 4–15. The children and their parents were both invited and on arrival, they filled in a structured questionnaire which recorded the demographic data, pharmacological treatment, coagulopathies if presented in the patient or his close family, family history of autoimmune diseases and family history of ADHD or hyperactivity.

#### *Healthy Children*

The blood samples were drawn from healthy children undergoing elective surgery (mainly inguinal hernias and undescended testis). The picked age range was similar to previously mentioned group and in this group, the same structured questionnaire was filled by the parents. These children had no major illness or knowledge of a recent infectious disease. The blood was drawn following full sedation of the child prior to commencing the surgery. The child's parent signed a consent form and filled in the same questionnaires that the previous group was presented.

### Methods

Approximately, 5 ml of blood was drawn from each subject for the following laboratory analysis; quantitative levels of immunoglobulins (class G and M) of anti-cardiolipin (aCL) antibodies, beta-2-glycoprotein I (β2GPI) antibodies, anti-phosphatidylserine antibodies and anti-ethanolamine antibodies. IgG and IgM class antibodies to anti-cardiolipin antibodies and β2GPI antibodies were measured by a commercial enzyme immunoassay (Orgentec Diagnostika Gmdh, Mainz, Germany) according to the manufacturer's instructions. For aCL, a value above 10 GPL for IgG and a value above 7 MPL for IgM were considered as "positive". For β2GPI, values greater then 8 units/ml are considered as "positive" and values 5–8 are considered "low positive" for IgG and IgM. Anti-ethanolamine antibodies and anti-phosphatidylserine antibodies were detected using ELISA. Microtitre plates (Costar Medium Binding EIA/RIA plates; Costar, Cambridge, MA, USA) were coated with 100 μl/well of phosphatidylserine and ethanolamine. After incubation with 120 μl/well of 10% fetal bovine serum (Sigma) in TBS for 2 h at room temperature the plates were washed three times with TBS. Then 100 μl/well of standards and serum samples diluted 1:100 in TBS were added in duplicate and the plates were incubated in the refrigerator (4°c) for the night. The plates were washed three times with TBS, and 100 μl/well of alkaline phosphatase-conjugated goat anti-human IgG or IgM (ACSC, Wesstbury, NY, USA) diluted 1:1000 TBS was added. After 2 h of incubation at room temperature, the plates were washed three times with TBS and 100 μl/well of *p*-nitrophenyl phosphate (Sigma) dissolved at 1 mg/ml in 1 M diethanolamine buffer (pH 9.8) was added. After 15 min the wells optic density were read by an optic reader at 450 nm wave length.

### Statistical Analysis

All the information gathered from the questionnaires was entered into an electronic database. All the statistical analyses were preformed using the BMDP computer program. ANOVA test was used to compare between the continuous variables in the two groups. The non-continuous variables were analyzed using Fisher's exact test and Pearson's Chi-Square where appropriate.

### RESULTS

The study included 70 children. Table I demonstrates the demographic characteristics of each group. In the ADHD group the age range is 5–15. The control group has an age range of 4–13. There is no statistical difference ($P = 0.001$) in the mean age of the two study groups. Forty eight percent of ADHD parents testified to a hyperactivity trait in a first degree relative of the tested case. Only 3.5% of the control parents testified to a similar trait. The two groups have similar percentages of positive answers in the other questions raised in the questionnaire: hypercoagulability in the close family, an autoimmune disease in the close family and a known coagulation abnormality in the tested case.

### aCL Levels

The mean level of these antibodies in the two groups is of no significant statistical difference as demonstrated in Fig. 1. A "positive" level (above 10 GPL units/ml for IgG and above 7 MPL units/ml for IgM) of these antibodies was measured for IgG in 12.2 and IgM in 26.8% of the ADHD children in comparison to 20 and 25%, respectively in the control group.

### Anti-B2GP Levels

The mean level of these antibodies in the two groups is of no significant statistical difference as demonstrated in Fig. 2. A "positive" level (above 8 units/ml for IgG and IgM) of these antibodies was measured for IgG in 4.8 and IgM in 2.4% of the ADHD children in comparison to 0 and 5%, respectively, in the control group.

### Anti-ethanolamine Levels

The mean level of these antibodies in the two groups is of no significant statistical difference as demonstrated in Fig. 3. A level of antibodies which is two standard deviations from the mean was found in 2.4% (1/41) of the ADHD children for the immunoglobulin G and M classes. As for the control group a level of two standard deviations from the mean was found in 3.6% (1/28) of blood samples for the immunoglobulin G class and in 10.7% (3/28) of the samples for the immunoglobulin M class.

TABLE I Main characteristics of the study population

| | ADHD group | Control group |
|---|---|---|
| Number of participants | 41 | 29 |
| Males | 90.2% ($N = 37$) | 75.9% ($N = 22$) |
| Females | 9.8% ($N = 4$) | 24.1% ($N = 7$) |
| Mean age | 9.98 | 7.76 |
| Methylphenidate (Ritalin) takers | 23 | 0 |
| Cloniride takers | 3 | 0 |

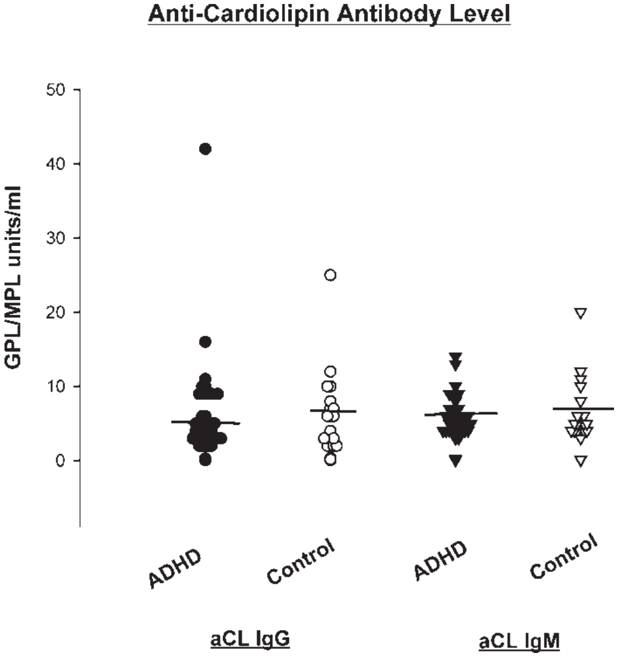

FIGURE 1 Comparison of IgG and IgM anti-cardiolipin antibody values in ADHD children and in a healthy control. The horizontal lines indicate means of pooled data for each group.

### Anti-phosphatidylserine Levels

The mean level of these antibodies in the two groups is of no significant statistical difference as demonstrated in Fig. 4. A level of antibodies which is two standard deviations from the mean was found in 2.4% (1/41) of the ADHD children for the immunoglobulin G and M classes. As for the control group a level of two standard deviations from the mean was not found in any blood sample for the immunoglobulin G class and in 10.7% (3/28) of

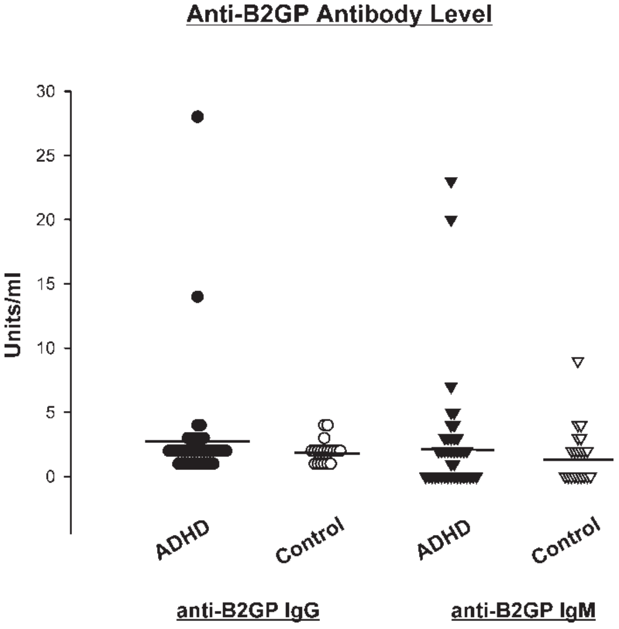

FIGURE 2 Comparison of IgG and IgM anti-β2GP antibody values in ADHD children and in a healthy control. The horizontal lines indicate means of pooled data for each group.

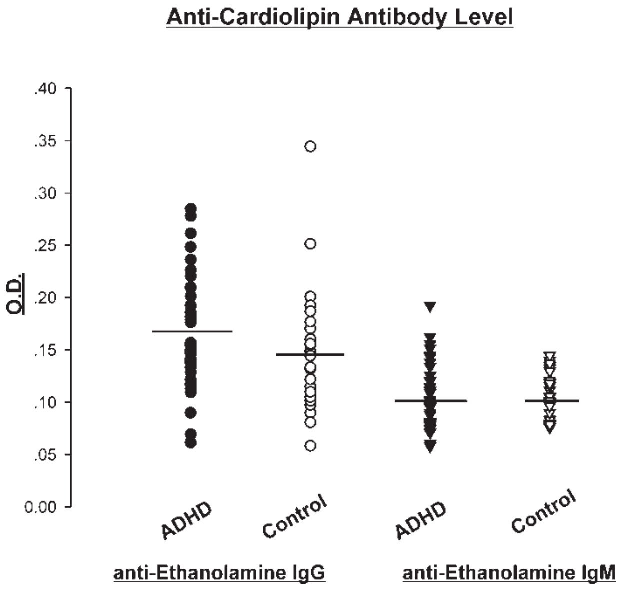

FIGURE 3 Comparison of IgG and IgM anti-ethanolamine OD values in ADHD children and in a healthy control. The horizontal lines indicate means of pooled data for each group.

the samples for the immunoglobulin M class. This latter group had no other "positive" antibodies and their parents did not report of any family history of coagulopathies, autoimmune disease or hyperactivity.

### DISCUSSION

This study tried to prove a postulated association between the presence of APLA in children's blood and the diagnosis of ADHD in those children. The assumption that such a connection exists relied on the appearance of neurological manifestations mainly in APS which are not fully explained by the procoagulopathic effect these antibodies induce, the *in vivo* effect and *in vitro* effect these antibodies have on nerve tissue and the hyperactivity induced on mice when these antibodies were injected to them. Our study results do not demonstrate an increased incidence of anticardiolipin antibodies, β2GP antibodies, anti-phosphatidylserine antibodies and anti-ethanolamine antibodies in ADHD children when compared to a similar group of healthy children. The percentage of children "positive" to the presence of anticardiolipin antibodies and β2GP antibodies in our study did not differ from that found in a healthy age matched population as studied by Avcin *et al.* (2001). The prevalence of the other two antibodies (anti-ethanolamine and anti-phosphatidylserine) in a healthy population was not studied from a review of the recent literature.

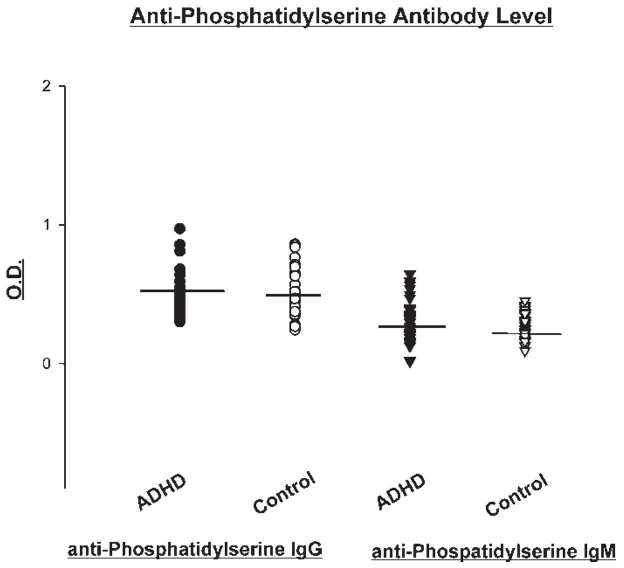

FIGURE 4 Comparison of IgG and IgM anti-phosphatidylderine OD values in ADHD children and in a healthy control. The horizontal lines indicate means of pooled data for each group.

A connection between APLA and neurological manifestations in children was demonstrated in a number of studies. Eriksson *et al.* (2001), in a study, similar to ours, found high prevalence of APLA in children with epilepsy (44% of the studied population). They also observed a significant variation in the prevalence of aCL in different epileptic syndromes. Yoshimura *et al.* (2001) found high prevalence of APLA in children with benign infantile convulsions when compared to an aged matched control (88% compared to 17%). A follow up on these children for two years disproved a number of prior assumptions: the first, in one-third of the children, high levels of aCL were detected even though they had not received any anti-epileptic medication and the antibody level decreased as expected with age without relation to the dose of drugs or term of therapy; the second, the frequency of convulsions did not influence the level of aCL; the third, the cross-reaction between aCL and neurons was more likely than the thrombogenic activity involved in APS as MRI findings were normal. Since migraine seems to be more common in patients with SLE, some studies have investigated a possible association with aCL (Markus and Hopkinson, 1992), and studies in adults with migraines have suggested a significant relationship between aCL and migraines (Shuaib *et al.*, 1989; Tietjen *et al.*, 1998). Verrotti *et al.* (2001) studied this relationship in children and adolescents and found no association.

The look for a single cause in syndromes is in times a very complicated task and in others a futile one. It is especially true in ADHD, where the diagnosis is not always made by an objective person and the disorder withholds a number of subclasses. From the information gathered to this date, it is believed that ADHD is a condition caused by a complex interaction of biological and environmental factors. Hyperactivity is known to aggregate within families for 25 years and was proved once more in our study. Twin, family and adoption studies, have consistently shown that hyperactivity is one of the most commonly inherited behavioral traits in childhood. The exact genetic defect or defects are not known. Current genetic research based on neurophysiological findings is centered on genes involved in the dopaminergic and noradrenergic function. Animal as well as human studies have shown the importance these neurotransmitters have in the development of hyperactivity (Todd and Botteron, 2001). The review by Biederman and Spencer (2000) emphasizes that an induced change in these neurotransmitter systems in children with ADHD affects their behavior and can improve their symptoms. A recent study by Segman *et al.* (2002) demonstrates a possible connection between the immune and these two neurotransmitters. They studied 86 families with children diagnosed with ADHD and found a significant association between a variation in the gene for the immune system protein interleukin-1 (IL-1) and ADHD. IL-1 has a pivot role in our immune system but also in the release of dopamine and noradrenalin in different brain areas.

While genetic studies showed a genetic contribution to this disorder, they also demonstrated that the environment played a role as well. The twin study by Goodman and Stevenson (1989) has concluded that heritability accounted for 30–40% of the cases, environmental factors accounted for 10–30% of the cases and 40–50% was accounted for by specific environmental effects and measurement error. The factors that have been shown to encourage the appearance of ADHD are those environmental factors that affect the ability of the organism to function or develop at a normal level. Initial studies centered on dietary substances that can influence the appearance of hyperactivity. However, in recent years additional environmental factors have been found across many areas including: toxins, allergies to dietary substances and seasons of birth. Those ADHD studies that could control best for possible confounding effects from the environment got better results.

In sum, our study has shown no increased incidence of APLA in children with ADHD compared with control subjects. The assumption that such a relationship exists relied on laboratory, animal and human studies that have demonstrated the influence these antibodies have on the single neuron, the entire nervous system and human behavior. The hypothesis that APLA can play a role in the etiopathogenesis of ADHD in children should be tested in larger studies if the association is to be proved.

### References

American Psychiatric Association (1994) *Diagnostic and Statistical Manual of Mental Disorders*, 4th Edn. (American Psychiatric Association, Washington DC).

Avcin, T., Ambrozic, A., Kuhar, M., *et al.* (2001) "Anticardiolipin and anti-B2 glycoprotein I antibodies *in sera* of 61 apparently healthy children at regular preventive visits", *Rheumatology* **40**, 565–573.

Biederman, J. and Spencer, T.J. (2000) "Genetics of childhood disorders: XIX. ADHD, part 3: Is ADHD a noradrenergic disorder?", *J. Am. Acad. Child. Adolesc. Psychiatry* **39**, 1330–1333.

Brey, R.L., Escalante, A., Futrell, N. and Asherson, R.A. (1996) "Cerebral thrombosis and other neurological manifestations in the antiphospholipid syndrome", In: Adherson, R.A., Cervera, R., Piette, J.C. and Shoenfeld, Y., eds, *The Antiphospholipid Syndrome* (CRC Press, Boca Raton), pp 133–146.

Cantwell, D.P. (1996) "Attention deficit disorder: a review of the past 10 years", *J. Am. Acad. Child. Adolesc. Psychiatry* **35**, 978–987.

Caronti, B., Pittoni, V., Palladini, G. and Valesini, G. (1998) "Anti-B2-glycoprotein I antibodies bind to central nervous system", *J. Neurol. Sci.* **156**, 211–219.

Chapman, J., Cohen-Armon, M., Shoenfeld, Y. and Korczyn, A.D. (1999) "Antiphospholipid antibodies permeabilize and depolarize brain synaptoneurosomes", *Lupus* **88**, 127–133.

Elia, J., Ambrosini, P.J. and Rapaport, J.L. (1999) "Treatment of attention deficit hyperactivity disorder", *N. Engl. J. Med.* **340**, 780–788.

Eriksson, K., Peltola, J., Keranen, T., *et al.* (2001) "High prevalence of antiphospholipid antibodies in children with epilepsy: a controlled study of 50 cases", *Epilepsy Res.* **46**, 129–137.

Goodman, R. and Stevenson, J. (1989) "A twin study of hyperactivity-II. The aetiological role of genes, family relationships, and perinatal adversity", *J. Child Psychol. Psychiatry* **30**, 691–709.

Hojnik, M., George, J., Ziporen, L. and Shoenfeld, Y. (1996) "Heart valve involvement (Libman-Sacks endocarditis) in the phospholipid syndrome", *Circulation* **93**, 1579–1587.

Hughes, G.R.V. (1983) "Thrombosis, abortion, cerebral disease and lupus anticoagulant", *Br. Med. J.* **187**, 1088–1089.

Kent, M.N., Alvarez, F.J., Vogt, E., *et al.* (1997) "Monoclonal antiphospholipid antibodies react directly with feline and murine central nervous system", *J. Rheumatol.* **24**, 1725–1733.

Kent, M., Alvarez, F.J., Ng, A.K. and Rote, N.S. (2000) "Ultrastructural localization of monoclonal antiphospholipid antibody binding to rat brain", *Exp. Neurol.* **163**, 173–179.

Markus, H.S. and Hopkinson, N. (1992) "Migraine and headache in systemic lupus erythematosus and their relationship with antibodies against phospholipids", *J. Neurol.* **239**, 39.

Robinson, L.M., Sclar, D.A., Skaer, T.L. and Galin, R.S. (1999) "National trends in the prevalence of attention-deficit/hyperactivity disorder and the prescribing of methylphenidate among school-age children", *Clin. Pediatr.* **38**, 209–217.

Segman, R.H., Meltzer, A., Gross-Tsur, V., *et al.* (2002) "Preferential transmission of interleukin 1 receptor antagonist alleles in attention deficit hyperactivity disorder", *Mol. Psychiatry* **7**, 72–74.

Shuaib, A., Barklay, L., Lee, M.A. and Suchowersky, O. (1989) "Migraine and antiphospholipid antibodies", *Headache* **29**, 42.

Tietjen, G.E., Day, M., Norris, L., *et al.* (1998) "Role of anticardiolipin antibodies in young persons with migraines and transient focal neurological events", *Neurology* **50**, 1433.

Todd, R.D. and Botteron, K.N. (2001) "Is attention-deficit/hyperactivity disorder an energy deficiency syndrome?", *Biol. Psychiatry* **50**, 151–158.

Verrotti, A., Cieri, F., Pelliccia, P., *et al.* (2001) "Lack of association between antiphospholipid antibodies and migranes in children", *Int. J. Clin. Lab. Res.* **30**, 109–111.

Yoshimura, K., Konishi, T., Kotani, H., *et al.* (2001) "Prevalence of positive anticardiolipin antibodies in benign infantile convulsion", *Brain Dev.* **23**, 317–320.

Ziporen, L., Shoenfeld, Y., Levy, Y. and Korczyn, A. (1997) "Neurological dysfunction and hyperactive behavior associated with antiphospholipid antibodies", *J. Clin. Investig.* **100**, 613–619.

